# Editorial: Advances in Pathogenesis and Therapies of Gout

**DOI:** 10.3389/fimmu.2022.890204

**Published:** 2022-04-01

**Authors:** Lihua Duan, Jixin Zhong, Ye Yang, Xiaoxia Zhu

**Affiliations:** ^1^ Department of Rheumatology and Clinical Immunology, Jiangxi Provincial People’s Hospital, The First Affiliated Hospital of Nanchang Medical College, Nanchang, China; ^2^ Department of Rheumatology and Immunology, Tongji Hospital, Tongji Medical College, Huazhong University of Science and Technology, Wuhan, China; ^3^ Department of Medicine, University of Florida, FL, Gainesville, United States; ^4^ Division of Rheumatology, Huashan Hospital, Fudan University, Shanghai, China

**Keywords:** gout, inflammation, inflammasome, hyperuricemia, monosodium urate

Gout is one of the most common metabolic disorders in human caused by inflammatory responses to the deposition of monosodium urate (MSU) crystals, which form in the presence of increased urate concentrations. The pathogenesis of gout is that MSU crystal triggers a strong inflammatory response by activating macrophages in tissues and promoting the collection of neutrophils to tissues or organs (1). It has been reported that many soluble mediators are implicated in the initiation and amplification of the gout flare, including pro-inflammatory cytokines, lipid mediators, and complement (2). However, activation of the NOD-, LRR- and pyrin domain-containing protein 3 (NLRP3) inflammasome by monosodium urate crystals with release of IL-1β plays a major role in the initiation of the gout flare (3). Interestingly, the gout flare is a self-limiting inflammation, and several mechanisms of resolution have been proposed, such as neutrophil extracellular traps (4), negative regulators of inflammasome and TLR signaling, and anti-inflammatory cytokines (5). It is noteworthy that gout is closely related to many comorbidities (6), especially cardiovascular diseases. To further our understanding of inflammatory regulation in the molecular pathophysiology of gout and to explore potential therapeutic approaches, this Research Topic exhibits a number of original research articles and review papers on the topic of advances in pathogenesis and therapies of gout.

In this Research Topic, Zhang et al. investigated the nationwide prevalence of hyperuricemia in China and evaluate its trends and associated risk factors. And significant escalating trends were observed between 2015-16 and 2018-19. Qiu et al. performed a series of bioinformatics analyses to identify molecular mechanisms related to gout, and found that pro-ADM can be used as a new inflammation related biomarker to predict and diagnose gout. Advanced imaging technology enables early gout diagnosis and can be used to evaluate the therapeutic effect. Li et al. summarized the role of ultrasonography, dual-energy computed tomography,and magnetic resonance imaging in the management of gout.

The activation of NLRP3 inflammasome and subsequent induction of the release of IL-1β exerts a central role in the initiation of gout flares. Besides MSU, various purine metabolites bind to different purine receptors for regulating IL-1β secretion implicated in the pathogenesis of gout flares (7). Given that the purine signaling pathway exerts different regulatory effects on inflammation, Tao et al. reviewed the role of purinergic receptor-mediated signaling pathways in the regulation of gout flare and resolution. The possibility that epigenetic mechanisms may contribute to gout pathogenesis offers the potential of a set of completely different therapeutic options (8). Georgel and Georgel reviewed the current mechanistic understanding of several components provided by food intake that are capable of modulating inflammation through epigenetic modification/reprogramming of innate cells.

Both psoriasis and gout are associated with increased risk of cardiovascular diseases (CVD) (9, 10), Chen et al. reported in this Research Topic that gout augments the risk of CVD independently of traditional risk factors in patients with psoriasis. This finding suggests that the management of gout/hyperuricemia may be beneficial in reducing the risk of developing CVD in patients with Psoriasis.

Galectin-9 (Gal-9) is a modulator of innate and adaptive immunity with both pro- and anti-inflammatory functions, dependent upon its expression and cellular location (11). Using mouse models of gout, Mansour et al. investigated the action of exogenous Gal-9 in MSU-gouty inflammation, which provide a new therapeutic strategy for preventing tissue damage in gouty arthritic inflammation. El Sayed et al. examined the anti-inflammatory mechanism of proteoglycan 4(rhPRG4) in MSU stimulated monocytes by animal model, Their work indicates that rhPRG4 exerts an anti-inflammatory activity in gout PBMCs, mediated by its ability to reduce urate crystal phagocytosis. Si-Miao-San (SMS)is a traditional Chinese medicine that has been reported to relieve the symptoms of gouty arthritis (12). A research by Cao et al. explored the anti-inflammatory mechanism of SMS on gout through animal and cellular experiments. Uric acid is excreted mainly through the kidneys and intestines. A mini review by Yin et al. summarized the effects of intestinal uric acid transporters and intestinal flora on uric acid excretion.

Collectively, the original research and review articles in this Research Topic cover a series of important aspects in the field of inflammatory regulation and clinical management in gout which may provide new insights into the diagnosis and treatment of gout and hyperuricemia.

## Author Contributions

All authors listed have made a substantial, direct and intellectual contribution to the work, and approved it for publication.

## Funding

This work was supported by grants from National Natural Science Foundation of China (81960296 and 81871286), Jiangxi Provincial Clinical Research Center for Rheumatic and Immunologic Diseases (20192BCD42005), and Outstanding Innovation Team of Jiangxi Provincial People’s Hospital (no. 19-008).

## Conflict of Interest

The authors declare that the research was conducted in the absence of any commercial or financial relationships that could be construed as a potential conflict of interest.

## Publisher’s Note

All claims expressed in this article are solely those of the authors and do not necessarily represent those of their affiliated organizations, or those of the publisher, the editors and the reviewers. Any product that may be evaluated in this article, or claim that may be made by its manufacturer, is not guaranteed or endorsed by the publisher.
